# Can OpenEHR, ISO 13606, and HL7 FHIR Work Together? An Agnostic Approach for the Selection and Application of Electronic Health Record Standards to the Next-Generation Health Data Spaces

**DOI:** 10.2196/48702

**Published:** 2023-12-28

**Authors:** Miguel Pedrera-Jiménez, Noelia García-Barrio, Santiago Frid, David Moner, Diego Boscá-Tomás, Raimundo Lozano-Rubí, Dipak Kalra, Thomas Beale, Adolfo Muñoz-Carrero, Pablo Serrano-Balazote

**Affiliations:** 1 Data Science Unit Hospital Universitario 12 de Octubre Madrid Spain; 2 ETSI Telecomunicación Universidad Politécnica de Madrid Madrid Spain; 3 Medical Informatics Unit Hospital Clinic de Barcelona Barcelona Spain; 4 Veratech for Health Valencia Spain; 5 The European Institute for Innovation through Health Data Gent Belgium; 6 Ars Semantica London United Kingdom; 7 Telemedicine and Digital Health Research Unit Instituto de Salud Carlos III Madrid Spain

**Keywords:** electronic health records, FAIR principles, health information standards, HL7 FHIR, ISO 13606, OpenEHR, semantics

## Abstract

In order to maximize the value of electronic health records (EHRs) for both health care and secondary use, it is necessary for the data to be interoperable and reusable without loss of the original meaning and context, in accordance with the findable, accessible, interoperable, and reusable (FAIR) principles. To achieve this, it is essential for health data platforms to incorporate standards that facilitate addressing needs such as formal modeling of clinical knowledge (health domain concepts) as well as the harmonized persistence, query, and exchange of data across different information systems and organizations. However, the selection of these specifications has not been consistent across the different health data initiatives, often applying standards to address needs for which they were not originally designed. This issue is essential in the current scenario of implementing the European Health Data Space, which advocates harmonization, interoperability, and reuse of data without regulating the specific standards to be applied for this purpose. Therefore, this viewpoint aims to establish a coherent, agnostic, and homogeneous framework for the use of the most impactful EHR standards in the new-generation health data spaces: OpenEHR, International Organization for Standardization (ISO) 13606, and Health Level 7 (HL7) Fast Healthcare Interoperability Resources (FHIR). Thus, a panel of EHR standards experts has discussed several critical points to reach a consensus that will serve decision-making teams in health data platform projects who may not be experts in these EHR standards. It was concluded that these specifications possess different capabilities related to modeling, flexibility, and implementation resources. Because of this, in the design of future data platforms, these standards must be applied based on the specific needs they were designed for, being likewise fully compatible with their combined functional and technical implementation.

## Problem to Solve: Electronic Health Record Standards Are Applied for Purposes for Which They Were not Designed

The electronic health record (EHR) is defined as the repository of health data generated throughout the patient’s lifetime, which is used in the provision of health care to the individual or the population [1]. Additionally, EHR data may have uses other than health care practice, known as secondary use, including activities such as health research or the evaluation of health outcomes [2]. In order to achieve a genuine use of EHR data, according to findable, accessible, interoperable, and reusable (FAIR) principles [3], it is necessary for information systems to overcome a number of shortcomings: (1) they are designed based on the generation of clinical reports where unstructured data predominates; (2) they embed the semantics of health domain concepts in the persistence data model; and (3) they do not apply health information standards or do so to a limited scope. One approach to solving these challenges lies in the design of health data platforms based on standards [4].

In this regard, different projects have emerged in Spain with the aim of building standardized EHR platforms. At a regional level, 2 projects aim to implement standardized persistence EHRs: the project launched in the Catalonia region [5], based on the OpenEHR specification [6], and the collaborative project between the regions of Castilla La Mancha and the Canary Islands [7], which is based on the International Organization for Standardization (ISO) 13606 standard [8]. At the national level, the Spanish Ministry of Health is leading a project for the exchange of EHR extracts across the different regions based on ISO 13606 [9], in contrast to previous national projects of other European countries such as Norway and Denmark based on OpenEHR [10,11]. Additionally, Spain participates in the European Patient Summary (EUPS) and International Patient Summary (IPS) initiatives [12,13], which use the Health Level 7 (HL7) Clinical Document Architecture (CDA) and HL7 Fast Healthcare Interoperability Resources (FHIR) standards [14,15], respectively, both oriented to the cross-border exchange of summarized EHR extracts.

As can be observed, the selection of the EHR standards is not consistent across the different health data initiatives, applying them indistinctly in aspects such as persistence or exchange of data. This gives rise to several key discussion points to assist health data platform project decision teams, who may not be experts in the various EHR standards, in selecting and applying them [16]. Although previous studies have analyzed the interaction between EHR standards from a technical perspective [17], the advances produced in recent years in this field, which have led to new standardization specifications and advanced uses of data, call for a new review that responds to:

- Which are the specific capabilities of today’s leading EHR standards?
- Which EHR standard should be selected and applied to next-generation data platforms and spaces?
- Are there successful implementations of standards-agnostic use of EHR standards?

For this reason, a panel of experts in EHR standards was formed to address these 3 key issues for designing health data platforms based on EHR standards. This panel is composed of 8 Spanish and 2 international experts with a long history of leading digital transformation in their respective organizations and a multidisciplinary approach: 2 managers of a tertiary hospital and a European institution for health data innovation (medical doctors specializing in health information management), 2 heads of digital health research groups (engineers), and 6 senior consultants in the development and implementation of EHR standards (engineers and medical doctors specializing in medical informatics). Therefore, this viewpoint aims to establish a coherent, agnostic, and homogeneous framework for the use of the most impactful EHR standards in the new-generation health data spaces: OpenEHR, ISO 13606, and HL7 FHIR.

## Analysis of EHR Standards Based on Detailed Clinical Models

Most health information systems are designed using single-model methodologies, in which the health domain concepts are embedded in the data model. In scenarios characterized by complexity, with a large number of concepts and a high tendency to change, systems based on this methodology are inflexible, expensive to maintain, and generally have to be replaced after a few years. The Detailed Clinical Models (DCM) paradigm, also known as dual-model methodology or 2-level modeling, provides a solution to the problems of the evolution and maintenance of health information systems [18]. On the one hand, it defines a reference model with the necessary components and their constraints to build a standard EHR. On the other hand, it establishes an archetype model for the formalization of the clinical-domain concepts according to the reference model. This paradigm allows separating knowledge (health concepts that are valid for all instances and that can evolve over time) and information (specific and immutable instances of health concepts), making the extension of the concept model flexible and software-independent [19]. Hence, having formally defined information models built from common components and linked to standard terminologies for a complete semantic representation [20], the meaning of the data can be interpreted without previous agreement, thus achieving interoperability and data reuse without loss of meaning or context [21]. The most relevant DCM-based standards in the current state of the art are the following:

- OpenEHR: It aims to create a standard EHR specification based on the dual-model methodology [6]. The reference model offers the components EHR, folder, composition, section, and entry and categorizes entries into observations, evaluations, instructions, and actions [22]. OpenEHR offers a platform model with services related to data entry, querying, persistence, and versioning. Additionally, it has a repository of over 880 archetypes, which includes around 10,000 clinical data points, making it the largest open repository of clinical models in the world [23].
- ISO 13606: It is a standard based on DCMs that enables the full-meaning exchange of EHR extracts. It consists of 5 parts, being the core of the standard part 1 “reference model” [8], and part 2 “archetype model” [24]. Its reference model defines the components: EHR extract, folder, composition, section, entry, cluster, and element. Likewise, parts 3, 4, and 5 define, respectively, the reference archetypes, security aspects, and communication interfaces [25-27].
- HL7 FHIR: It provides a standard framework for the agile development of health data exchange processes [15]. This specification is inspired by the dual-model paradigm, providing a predefined catalog of information models, denominated “resources.” These are designed mainly at the clinical entry-level and can be grouped into bundles, referenced from compositions, refined through extensions, constrained into profiles, and transmitted through web services and messages.

To understand the strengths and weaknesses of these standards, the panel of experts has analyzed the capabilities of the above standards in several web-based sessions, thus answering the question, “Which are the specific capabilities of today’s leading EHR standards?” First, a set of common points to be studied and compared between OpenEHR, ISO 13606, and HL7 FHIR were identified, according to the inconsistencies observed in the EHR platforms currently under implementation [5,7,9,12,13]. These points of analysis correspond to aspects of (1) purpose design, (2) modeling capabilities, (3) application flexibility, and (4) implementation resources offered. The agreed points were then independently studied by each expert based on the documentation provided by the standards and then discussed together until a common position on the capability of each standard was agreed upon.

Some insights were drawn from this discussion. In terms of design (analysis points D1-D4 in Table 1), OpenEHR specification provides a comprehensive platform model for data recording, persistence, and querying [6,22,28,29], while ISO 13606 and HL7 FHIR must be supported by external developments for these services [8,30]. Likewise, ISO 13606 and HL7 FHIR provide common frameworks for the exchange of information extracts through their messaging components and communication interfaces [24,27,31], while OpenEHR focuses on on-demand data extraction and retrieval [32]. In terms of modeling capabilities (M1-M3 in Table 1), both OpenEHR and ISO 13606 allow modeling and formalization of knowledge (clinical-domain concepts) through their reference models and archetypes [6,24], while HL7 FHIR offers limited functionality for building profiles from predefined resources [33]. In addition, all 3 specifications allow formalizing clinical documents and entries [6,8,34]. Regarding flexibility (F1-F3 in Table 1), although the 3 specifications allow specialization of already-created concepts, only OpenEHR and ISO 13606 allow building new concepts based on specific requirements [6,24]. Likewise, all 3 are flexible in the incorporation of terminological standards into the information models [24,35,36]. Finally, in terms of implemented resources (I1-I4 in Table 1), both OpenEHR and HL7 FHIR offer solutions, complete or in a limited way, for the information models catalog (eg, the international catalog of OpenEHR archetypes from the Clinical Knowledge Manager tool) [15,23], clinical decision support [37,38], data retrieval interfaces based on application programming interface [28,39], and data messaging [32,39]. In contrast, ISO 13606 does not offer implemented components beyond theoretical formal definitions of information models and data exchange interfaces [25-27].

Table 1 summarizes the comparative analysis, indicating, for each key point, “suitable,” when the panel agrees that the standard incorporates this capability natively; “limited,” when it is restricted or must be solved by an external development; and “inadequate,” when the standard, in its current state, cannot incorporate it.

**Table 1:** Summary comparative analysis of OpenEHR, International Organization for Standardization (ISO) 13606, and Health Level 7 (HL7) Fast Healthcare Interoperability Resources (FHIR) capabilities.

	OpenEHR	ISO 13606	HL7 FHIR
(D1) Design focused on EHR^a^ clinical recording	Suitable	Limited	Limited
(D2) Design focused on EHR persistence	Suitable	Limited	Limited
(D3) Design focused on EHR exchange	Limited	Suitable	Suitable
(D4) Design focused on EHR querying	Suitable	Limited	Limited
(M1) Modeling and formalization of clinical-domain concepts	Suitable	Suitable	Limited
(M2) Modeling and formalization of clinical documents	Suitable	Suitable	Suitable
(M3) Modeling and formalization of clinical entries	Suitable	Suitable	Suitable
(F1) Flexibility to create new concepts	Suitable	Suitable	Inadequate
(F2) Flexibility to specialize implemented concepts	Suitable	Suitable	Limited
(F3) Flexibility to incorporate terminological standards	Suitable	Suitable	Suitable
(I1) Implementation of information model catalog	Suitable	Limited	Limited
(I2) Implementation of CDSS^b^ component	Suitable	Limited	Limited
(I3) Implementation of API^c^ query component	Suitable	Limited	Suitable
(I4) Implementation of messaging component	Limited	Limited	Suitable

^a^EHR: electronic health record.

^b^CDSS: clinical decision support system.

^c^API: application programming interface.

## Proposal of Agnostic Guideline for the Selection and Application of EHR Standards

Once the strengths and weaknesses of each standard had been determined, the panel of experts set out to answer the question, “Which EHR standard should be selected and applied in next-generation data platforms and spaces?” Thus, with the analysis of 5 relevant EHR initiatives based on standards [5,7,9,12,13], a set of 11 common needs to be solved through the application of standards in data platforms were identified, grouped into 5 categories: modeling, persistence, exchange, query, and service implementation. These points were discussed jointly by the experts, and then, based on the previous analysis of the capabilities of the standards (Table 1) and the knowledge and experience of each member, some conclusions were reached.

Hence, it was agreed that OpenEHR is the only specification that provides a comprehensive solution for building a standardized EHR, as it offers a complete platform specification for knowledge modeling, recording, persisting, and querying health data, and it is supported by an active international community [6,23,29,37]. This key message is also supported by the numerous EHR solutions implemented that have incorporated this standard [40]. Likewise, ISO 13606 and HL7 FHIR have proven useful for data repository services [41-43], although their limitations for this purpose and the need for additional external developments for their suitability must be considered [8,30]. Regarding data exchange requirements, both ISO 13606 and HL7 FHIR can be used, depending on the complexity and capacity for agreement between parties. HL7 FHIR offers a minimum exchange framework, limiting flexibility for convergence and simplicity [31], whereas ISO 13606 offers a solution for semantic interoperability with the flexibility to adapt to heterogeneous EHR information models [24]. Therefore, ISO 13606 is preferred for complex interoperability projects such as regional, national, or international EHR interoperability initiatives [7,9], while FHIR is optimal for information systems integration processes in a single organization. Finally, OpenEHR and HL7 FHIR offer multiple implemented resources due to their active communities [23,44]. This is especially relevant for OpenEHR, which, through the Clinical Knowledge Manager tool, provides a rich catalog of clinical archetypes that is the result of a quality control review process.

Table 2 specifies the standards-agnostic usage guide for the different services of the health data platforms, as proposed by the expert panel.

**Table 2:** Standards-agnostic selection guide for providing health data platform services.

Service	Health data platform service	Preferred standards
Modeling	Modeling and formalization of clinical-domain concepts	OpenEHR and ISO^a^ 13606^b^
Persistence	Detailed and multipurpose data persistence	OpenEHR
Exchange	Complex and full-meaning data exchange	ISO 13606 and HL7^c^ FHIR^d,e^
Exchange	Simple and agile point-to-point data exchange	HL7 FHIR
Querying	Data query according to complex semantic restrictions	OpenEHR
Implementation	Design of data entry components in EHR^f^	OpenEHR
Implementation	EHR repository for clinical decision support processes	OpenEHR
Implementation	EHR repository for populating RWD^g^ repositories	OpenEHR
Implementation	Semantically interoperable platform for heterogeneous source EHRs	ISO 13606 and HL7 FHIR^b^
Implementation	Semantically interoperable exchange between EHR applications	HL7 FHIR
Implementation	Semantically interoperable exchange between EHR and RWD repositories	HL7 FHIR

^a^ISO: International Organization for Standardization.

^b^Limited implemented resources.

^c^HL7: Health Level 7.

^d^FHIR: Fast Healthcare Interoperability Resources.

^e^Limited flexibility.

^f^EHR: electronic health record.

^g^RWD: real-world data.

## Application of the Agnostic Approach in Health Data Platforms

For the validation of the proposed framework (Table 2), it is necessary to address the question, “Are there successful implementations of standards-agnostic use of EHR standards?” To this end, 3 relevant health data platforms based on the standards-agnostic approach proposed in this viewpoint, in which members of the panel of experts have participated, are described below.

**Figure 1:**
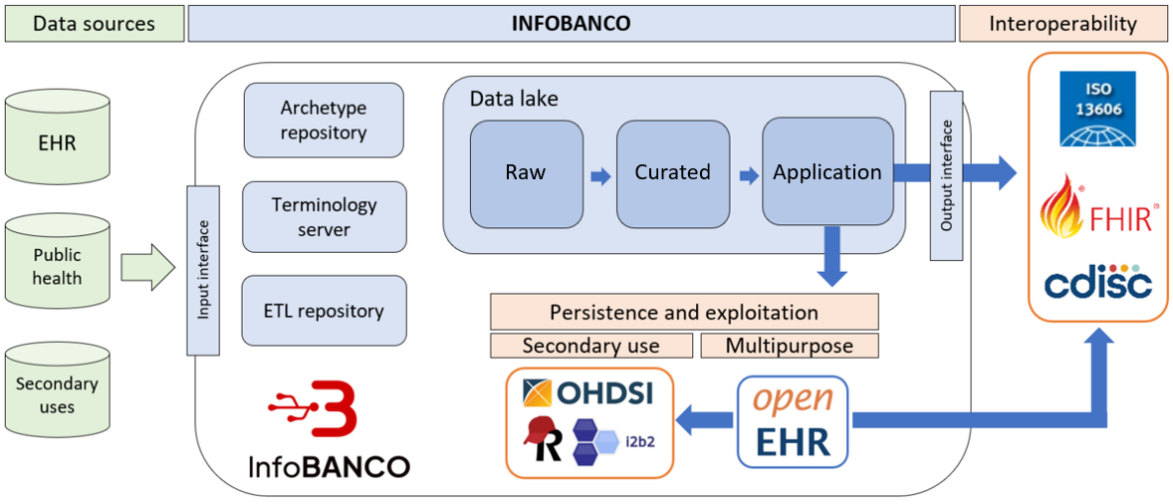
Architecture scheme of the INFOBANCO platform. EHR: electronic health record; ETL: extraction, transformation, and loading process.

First, the INFOBANCO platform, designed and implemented by the Data Science Unit of “12 de Octubre” Hospital in Madrid, Spain, constitutes a platform that offers health data management, persistence, query, and exchange services [45]. The interoperability interfaces include the previously mentioned standards HL7 FHIR and ISO 13606, as well as the Clinical Data Interchange Standards Consortium (CDISC) resources, which is used specifically in the clinical research field [46]. Besides, as persistence components, it implements a core OpenEHR repository based on Better Platform technology [47], along with others relying on standardized models for real-world data research, such as Informatics for Integrating Biology and the Bedside (i2b2) and Observational Medical Outcomes Partnership Common Data Model (OMOP CDM) [48,49]. The whole architecture of the platform, as well as the management of these standards, is supported by a raw data lake, an archetype server, a terminology server, and an extraction, transformation, and loading process server (Figure 1). Therefore, its design, agnostic to any specific standard, is based on the principle of applying each one to its intended design purpose. This results in an advanced data platform that offers multiple interoperability and analytical services, which are provided according to the needs of the specific use case [50,51].

Similarly, OntoCR is an ontology-based clinical repository for the registry and storage of structured data designed and implemented by the Medical Informatics Unit of “Clínic Barcelona” Hospital in Catalonia, Spain [41]. Besides the reuse of previously declared knowledge and the inference of new knowledge, the use of ontologies allows the modeling of information using any terminology, classification, and health information standard. To this end, an ontology must be created with the classes, metaclasses, and properties that define the standard, and then it is mapped to the variables defined in the local data model. Therefore, there is complete independence with respect to any specific standard, being able to carry out transformations between ISO 13606, OpenEHR, HL7 FHIR, and even standards for secondary use of data, such as OMOP CDM [52]. As an example, in the European project “Artificial Intelligence Supporting Cancer Patients Across Europe” (ASCAPE) [53], data related to daily step counts and adverse events coming from a mobile app were standardized under the ISO 13606 standard and then loaded into OntoCR. Hence, these EHR extracts could be translated to other reference models through semantic conversions based on the defined ontologies.

Finally, LinkEHR is a multireference model tool for the design and mapping of archetypes from legacy data and the model transformation between standards, widely used in the technical and scientific community [54]. This platform is completely based on the Archetype Object Model [55], which allows the tool to be able to work with any reference model, including ISO 13606, OpenEHR, HL7 CDA, HL7 FHIR, and CDISC. This method also enables the translation of archetypes between different reference models, providing full-meaning syntactic transformations, for example, OpenEHR archetype into ISO 13606 or HL7 FHIR standards. These transformations use a defined set of rules to convert semantically rich models into more generic ones, like the OpenEHR to ISO 13606 automatic transformation or the OpenEHR to HL7 FHIR semiautomatic transformation, which requires the user to make decisions to guide it. Finally, it also offers the possibility to export archetypes from any reference model into HL7 FHIR logical models, that is, a mechanism to represent clinical models based on other standards.

## Conclusions

In this viewpoint, a panel of experts in EHR standards has studied the problem of inconsistent application of EHR standards in health data projects, reaching a series of conclusions for the questions raised in the introduction of the work. First, the EHR standards analyzed have different characteristics of modeling, flexibility, and implementation resources (Table 1). For this reason, in the design of future data platforms, these specifications must be applied according to the diverse needs to be resolved related to information modeling, persistence, consultation, exchange, and implementation of services (Table 2). Finally, the agnostic application of these standards has been successfully applied to different health data platforms, demonstrating that they are fully compatible.

This work is not intended to replace technical studies on the combined use of EHR standards [56-58], but to offer a framework of recommendations to be applied in future initiatives on the design, implementation, and evaluation of health data platforms based on standards. This is essential in the current scenario of implementing the European Health Data Space, which advocates harmonization, interoperability, and reuse of data without regulating the specific standards to be applied for this purpose [59]. Thus, as final conclusions, we can affirm that OpenEHR, ISO 13606, and HL7 FHIR are useful for the purposes for which they have been designed and have limitations for those for which they have not been, being functionally and technically compatible for their joint implementation according to the need to be solved.

## Abbreviations

ASCAPE: Artificial Intelligence Supporting Cancer Patients Across Europe
CDA: Clinical Document Architecture
CDISC: Clinical Data Interchange Standards Consortium
DCM: Detailed Clinical Models
EHR: electronic health record
EUPS: European Patient Summary
FAIR: findable, accessible, interoperable, and reusable
FHIR: Fast Healthcare Interoperability Resources
HL7: Health Level 7
i2b2: Informatics for Integrating Biology and the Bedside
IPS: International Patient Summary
ISO: International Organization for Standardization
OMOP CDM: Observational Medical Outcomes Partnership Common Data Model
